# Elucidating Viral Communities During a Phytoplankton Bloom on the West Antarctic Peninsula

**DOI:** 10.3389/fmicb.2019.01014

**Published:** 2019-05-14

**Authors:** Tomás Alarcón-Schumacher, Sergio Guajardo-Leiva, Josefa Antón, Beatriz Díez

**Affiliations:** ^1^Department of Molecular Genetics and Microbiology, Pontificia Universidad Católica de Chile, Santiago, Chile; ^2^Max Planck Institute for Marine Microbiology, Bremen, Germany; ^3^Department of Physiology, Genetics, and Microbiology, University of Alicante, Alicante, Spain; ^4^Center for Climate and Resilience Research (CR2), University of Chile, Santiago, Chile

**Keywords:** phytoplankton blooms, Southern Ocean, viruses, environmental genomics, viral ecology

## Abstract

In Antarctic coastal waters where nutrient limitations are low, viruses are expected to play a major role in the regulation of bloom events. Despite this, research in viral identification and dynamics is scarce, with limited information available for the Southern Ocean (SO). This study presents an integrative-omics approach, comparing variation in the viral and microbial active communities on two contrasting sample conditions from a diatom-dominated phytoplankton bloom occurring in Chile Bay in the West Antarctic Peninsula (WAP) in the summer of 2014. The known viral community, initially dominated by Myoviridae family (∼82% of the total assigned reads), changed to become dominated by Phycodnaviridae (∼90%), while viral activity was predominantly driven by dsDNA members of the Phycodnaviridae (∼50%) and diatom infecting ssRNA viruses (∼38%), becoming more significant as chlorophyll *a* increased. A genomic and phylogenetic characterization allowed the identification of a new viral lineage within the Myoviridae family. This new lineage of viruses infects *Pseudoalteromonas* and was dominant in the phage community. In addition, a new Phycodnavirus (PaV) was described, which is predicted to infect *Phaeocystis antarctica*, the main blooming haptophyte in the SO. This work was able to identify the changes in the main viral players during a bloom development and suggests that the changes observed in the virioplankton could be used as a model to understand the development and decay of blooms that occur throughout the WAP.

## Introduction

The Southern Ocean (SO) is one of the world’s most productive marine regions. It plays a key role in carbon drawdown and contributes to an estimated 20% of global oceanic CO_2_ uptake (Takahashi et al., 2002). Seasonality in the SO leads to environmental variations; such as increased light availability and sea ice cover retreat along the summer, inputting metals and other nutrients to the surface waters and promoting water column stratification. The latter leads to large phytoplankton blooms (Vernet et al., 2008; Giovannoni and Vergin, 2012; Mendes et al., 2012), resulting in increased carbon uptake in coastal waters (Bunse and Pinhassi, 2017).

The West Antarctic Peninsula (WAP) blooms are acknowledged to be dominated first by large diatoms (>10 μm), followed then by smaller flagellates such as haptophytes and cryptophytes (Ducklow et al., 2012; Schofield et al., 2017). These groups modulate carbon sinking, microbial community composition, strongly influence higher trophic levels and support food webs (Arrigo, 1999; Piquet et al., 2011).

The influence of the phytoplankton bloom on bacterioplankton is well described in the SO, where chlorophyll *a* (Chl-*a*) concentration is positively correlated with bacterioplankton abundance (Luria et al., 2016; Evans et al., 2017). In Antarctic waters, some specific bacterial taxonomical groups increase their abundance in response to the presence of specific phytoplankton blooming species (Delmont et al., 2014). Thus, bacterioplankton production is limited by phytoplankton production, including species composition, bloom intensity, and duration. In turn, these phytoplankton features are limited by nutrient availability and regulated by biological interactions such as grazing intensity, and mainly viral activity (Riemann and Winding, 2001; Rooney-Varga et al., 2005; Delmont et al., 2014).

Viruses are among the most abundant and active biological entities in the ocean (Bergh et al., 1989; Brussaard et al., 2004). Through lytic virus–host interaction, host cellular content is channeled into the dissolved organic matter pool, changing the abundance of bacterial and eukaryotic hosts and influencing microbial community assemblage (Suttle, 2005). Thus, viral lysis is one of the major global drivers of phytoplankton bloom decay for diverse photosynthetic organisms in global oceans (Maranger et al., 1994; Lawrence and Suttle, 2004), however, little information is available on the viral effects on these blooms in the SO.

The virus to bacteria ratio (VBR) in Antarctic waters (0.7 to 6) is lower than the ocean average (1 to 50) (Wommack and Colwell, 2000), however, viral abundances in the SO present seasonal variations coupled with bacterioplankton and phytoplankton increase (Evans et al., 2017), and it has been suggested that viral lysis is more significant than bacterivory (Vaqué et al., 2017). The limited taxonomical analyses of viral communities in the SO indicate up to ∼90% of dsDNA viruses, from the order Caudovirales, dominate during the spring-summer transition (Brum et al., 2016). Apart from this, RNA viruses may make up an important part of the virioplankton, ranging from 8 to 65% of the total viral community in specific periods (Miranda et al., 2016). These are the only known studies to date that attempted to characterize, through genomics, viral communities in the SO. Despite this, there are no multi-omics studies elucidating changes in virioplankton composition and activity during a summertime phytoplankton bloom in Antarctic coastal waters.

Chile Bay (South Shetland Islands), is a representative site of coastal environments in the WAP where the activities and composition of the eukaryotic and prokaryotic communities have been previously studied during the austral summer (Alcamán-Arias et al., 2018; Fuentes et al., 2018). In particular, during the summer of 2014, metagenomic and metatranscriptomic measurements revealed changes in these communities represented by two contrasting samples of low and high chlorophyll periods in the waters of Chile Bay (Alcamán-Arias et al., 2018). Therefore, these two samples from the end of the summer in 2014 where a phytoplankton bloom occurred, give us an unbeatable scenario to study the composition and activity of the virioplankton community and their interactions with their bacterial and eukaryotic hosts.

Our analyses provide novel viral genomic information of the most relevant viruses that infect the most relevant microorganisms in this marine system, which provides a first view of the virus–host interactions that could shape the microbial communities within the WAP.

## Materials and Methods

### Sample Site and Physicochemical Data

Two samples (February 14th and March 4th) were collected at the Chile Bay (062°27′6°S; 059°40′6°W) (see Supplementary Figure 1), located in Greenwich Island (South Shetlands Islands, West Antarctic Peninsula) in 2014 (Alcamán-Arias et al., 2018). Sample collection as well as physicochemical and biotic parameters were previously described in Alcamán-Arias et al. (2018) and are now resumed in Supplementary Table 1. Briefly, subsurface seawater from 2 m depth, was pumped up through a 200 μm polyester net to exclude large organisms and particles and transported in darkness to the laboratory. Between 3 and 4 liters (for RNA and DNA, respectively) of seawater was filtered through 20 and 8 μm polycarbonate filters, and finally, 0.22 μm PES Sterivex filters (Millipore) using a Cole Palmer System peristaltic pump Model no. 7553-70 (6–600 rpm; pressure up to 2 bar). The RNA filters were preserved in RNA later and maintained at –80°C with the DNA filters until nucleic acids extraction and sequencing. Temperature, conductivity (salinity derived), and O_2_ concentration were measured *in situ* with a multiparameter sensor (OAKTON PCD650). PAR was measured with a HOBO Pendant Light, 64K- UA-002-64 sensor. Seawater samples for the determination of NO_2_^-^, NO_3_^-^, PO_4_^3-^ and Si(OH)_4_ nutrients were taken in triplicate, collected in 15-ml polyethylene flasks, and stored at –20°C until further analysis. The analysis was conducted using standard colorimetric techniques in a segmented flow Seal AutoAnalyser (Seal Analytical AA3 of four channels) (Armstrong et al., 1967) and silicic acid was measured according to the method of Thomsen et al. (1983).

For total Chl-*a* determination, 1 L of seawater from each sample was immediately filtrated by a glass fiber filter (GF/F), size pore 0.7 μm, and then stored at –20°C in darkness. For chlorophyll determinations, extractions with 10 mL of acetone (90% v v^-1^) for 20 h at –20°C in the dark were performed. Subsequently, fluorometric measurements using a 10AU Field and Laboratory Fluorometer (Turner Designs) and chlorophyll concentrations calculations were performed according to the protocol of Strickland and Parsons (1972), with chlorophyll *a* derivate from the equation:

$$\text{mgchlorophyll}\;\;a{\text{/m}}^{\text{3}}\;\text{=}\;F_{\text{D}}{\;}^{\text{*}}\;R\;\;\;\;\;\;\;\;\;\;\;\;\;\;\;(1)$$

where *R* is the fluorometric reading and *F* is a factor for each door (see Strickland and Parsons, 1972 for further specifications).

### Nucleic Acids Extraction and Metagenomes Sequencing

Total DNA and RNA were obtained from the 0.22 μm filters collected on February 14th and March 4th, which represent samples collected at low and high Chl-*a* content periods (L-C and H-C), respectively as described in Alcamán-Arias et al. (2018) and Fuentes et al. (2018). DNA and RNA extractions were performed with the method of Díez et al. (2001), as described in Alcamán-Arias et al. (2018). DNA and RNA were sequenced also as described in Alcamán-Arias et al. (2018) using Hi-seq technology (DNA Sequencing and Genotyping Center, DE, United States). Briefly, DNA libraries were prepared using the NEXTflex Rapid DNA-Seq Kit (Bioo Scientific Corporation, Austin, TX, United States) according to the manufacturer’s instruction. For metatranscriptomics, total RNA (500 ng) was cleaned up of rRNA prior to library construction by Ribo-Zero rRNA Removal Kit Bacteria (Illumina, San Diego, CA, United States) according to the manufacturer’s instruction followed by library construction using a NEXTflex Rapid Directional RNA-Seq kit (Bioo Scientific) according to the manufacturer’s instructions.

### Quality Assessment and Trimming

A quality assessment of metagenomic and metatranscriptomic data was performed as described in Alcamán-Arias et al. (2018) with the software FastQC (Andrews, 2010). Briefly, quality trimming was performed with the software PrinSeq (Schmieder and Edwards, 2011), with the following quality filters: a mean read quality of 30, a 3′ trimming for bases with quality below 30, a hard clipping of the first 7 leftmost bases for L-C metagenomic sample and the first 9 bases for H-C sample. For metatranscriptomes, a minimum quality of 30 and a minimum length of 30 bp was inquired, also a hard clipping of the first 11 bases of 5′ on both samples was performed. Low complexity sequences were filtered using the DUST model (Morgulis et al., 2006), implemented on Prinseq, with a threshold value of 7.

### Viral Mining

To recover viral sequences from total DNA and RNA samples, prior removal of sequences belonging to the three domains of life, Bacteria, Archaea and Eukarya was performed, through end-to-end mapping against the NCBI non-redundant (NR) database using Bowtie2 aligner (Langmead and Salzberg, 2012). Viral sequences were then recruited by end-to-end mapping against a viral database from RefSeq (Release 75) containing all complete viral genomes, excluding metazoan viruses because they were out of the focus of this study (non-animal virus database, NAV). Recruited viral sequences were assigned using Blastn (Camacho et al., 2009) against NAV database and then parsed and displayed using the lowest common ancestor algorithm implemented on MEGAN 6 (Huson et al., 2016). Overall taxonomic assignment of reads can be found in Supplementary Table 2.

### Viral Genome Recruitment and Functional Annotation

The two metagenomics samples were assembled separately but were also co-assembled using De Bruijn graphs implemented on Spades with the metagenomic mode (Bankevich et al., 2012) and Ray-meta assembler (Boisvert et al., 2012) with the default parameters, respectively. Only contigs over 200 bp were aligned against the NAV database using the multiple genome aligner Mauve (Darling et al., 2004), smaller contigs were discarded because they don’t reach the minimum length required by NCBI for annotation (Tatusova et al., 2016). Aligned contigs were reordered against individual genomes of related viruses from the NAV database. Cohesive endings form candidate contigs were checked using NUCmer (Delcher et al., 2002) with coverage 95% of similarity to avoid assembly bias due to k-mer early classification. Proteins were predicted from candidate genomes using a metagenomic version of Prodigal (Hyatt et al., 2010). Functional annotation was done with Interproscan 5 (Jones et al., 2014) and manually curated. Genome draft was plotted using CGview (Stothard and Wishart, 2005) and compared against a reference with blast visualizer Kablammo (Wintersinger and Wasmuth, 2014).

### Phylogenetic Analyses of Viral Molecular Markers and Statistical Analyses

For phylogenetic analyses of bacterial viruses, representative reference sequences of major capsid protein (MCP) from members of Myoviridae subfamilies and genera as well as unclassified members of this group were included in the analyses to elucidate the membership of potential novel viruses recovered. Also, a full genome alignment was performed between genomes retrieved in this work with all available viral genomes from nearest phylogenetic genera present in the database (e.g., *FelixO1* virus). Additional phage classification was verified using the VirFam platform (Lopes et al., 2014), with the Head-neck-tail module genes database.

On the other hand, DNA polymerase type B sequences from known members of Phycodnaviridae and extended Mimiviridae families, as well as sequences from the divergent Asfarviridae family all infecting eukaryotes, were included and analyzed, based on previous phylogenetic analyses (Yutin et al., 2015). Complete and partial gene sequences from environmental samples were also included. These sequences were aligned with partial DNA pol B phycodnavirus (PaV) predicted protein from the co-assembly. Both alignments were performed with MAFF (Katoh and Standley, 2013) using the G-INS-1 strategy and default parameters. Maximum likelihood trees were built using the IQTree (Trifinopoulos et al., 2016) with 10000 bootstraps and 10000 SH-aLRT branch tests. The substitution model that fits better in both cases with the data was LG (Le and Gascuel, 2008), with four gamma categories. A Bayesian model approximation was also applied using MrBayes (Ronquist et al., 2012) with the LG substitution model and 2.6 million generations for DNA pol B phylogenetic analysis. After the convergence, Markov Chain Monte Carlo (MCMC) results were summarized using TRACER (Rambaut et al., 2018) in order to ensure that all parameters had values > 2.

### Taxonomic Composition of Cellular Fractions

The cellular composition of the microbial community was assessed as described in Alcamán-Arias et al. (2018). Briefly, 16S rRNA gene sequences present in the metagenomes were identified and extracted using Ribopicker (Schmieder et al., 2012), then annotated using Blastn (Camacho et al., 2009) with Silva 123 SSU database (Quast et al., 2013) and displayed with MEGAN 6 (Huson et al., 2016). For the photosynthetic eukaryotic community composition, sequences matching chloroplast 16S rRNAs from 20–8 to 8–0.22 μm filter fractions sequenced with Illumina TAGs (iTAGs) from the study of Fuentes et al. (2018) were extracted, combined and reassigned against Phytoref database (Decelle et al., 2015), and analyzed for their relative abundance and composition. The activity of the phytoplankton community was assessed with total transcripts as well as metabolic genes such as rubisco (rbcL) and photosystem a (psbA) as described in Alcamán-Arias et al. (2018).

For the inference of eukaryotic and bacterial communities relationship with the physicochemical parameters, Spearman correlation coefficients were calculated using R and plotted with ggplot2 package (Wickham, 2017).

## Results

### Variations in the Viral Active Community Composition and Abundance

**Figure 1:**
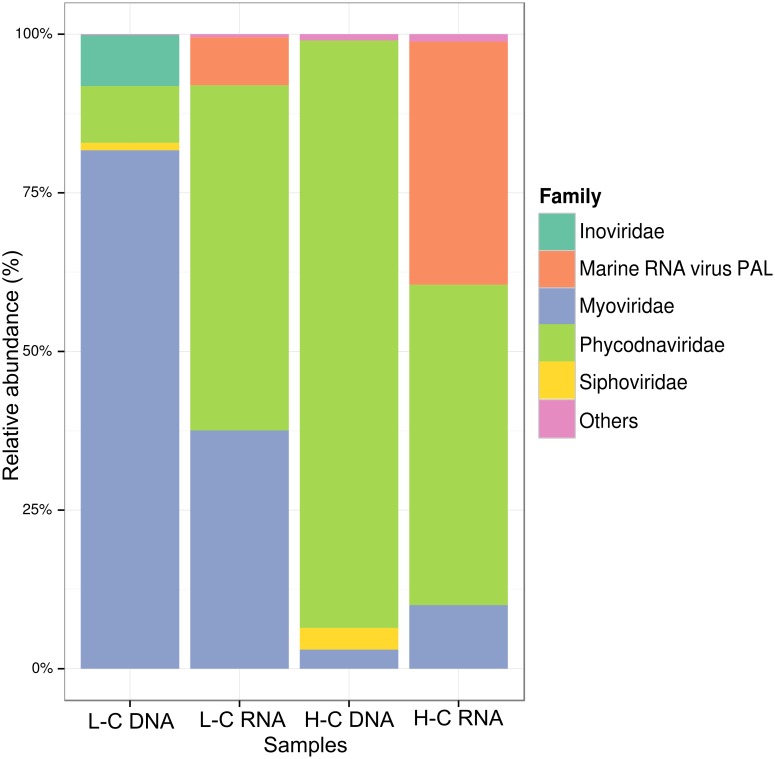
Taxonomic composition of viral communities in Chile Bay during a summer phytoplankton bloom in 2014. Relative abundances of all viral reads were retrieved by metagenomics and metatranscriptomics from two samples obtained under a low chlorophyll and a high chlorophyll period that were assigned against the NAV database. Only taxa with relative abundance > 1% are included, lower abundance were grouped into others category.

Combined metagenomics and metatranscriptomics data were used to analyze the viral community, revealing a markedly distinct composition and activity between the L-C and H-C contrasting summer time samples (Figure 1). The L-C metagenomic sample showed a viral community dominated by the order Caudovirales (that infects bacteria and archaea), with the Myoviridae family representing ∼82% of the total viral assigned reads, and the Siphoviridae family only representing ∼1%. Following, the Megavirales order held the second largest majority, represented only by the Phycodnaviridae family (9% of the total viral reads). Despite dsDNA viruses being dominant, bacterial filamentous ssDNA (+) viruses from the Inoviridae family represented the third most common viral group (∼8% of the total viral assigned reads). Metatranscriptomic analysis revealed that the most active viral group was the Phycodnaviridae family in the L-C sample (∼54% of the total viral assigned reads), followed by the Myoviridae family with a relative abundance of ∼38%. Furthermore, the presence and activity of sequences related to the marine RNA virus PAL E4 and PAL156, a group of ssRNA virus, with no DNA stage, were shown through the metatranscriptomic (∼8% combined in the L-C sample).

Although similar dsDNA viral families were also present in the H-C sample, their relative abundances changed radically. Here, the Phycodnaviridae family (∼93% of the total reads) from the Megavirales order was the dominant, and Caudovirales order presented a dramatic drop-off (Figure 1) showing a decrease in the Myoviridae family up to ∼3% of the total community, while the Siphoviridae family slightly increased to ∼3.5%. In the same way, the Inovirus group disappeared from the major taxa, showing a relative abundance of less than 1%. These pronounced changes in the metagenomic profiles between both types of summertime samples were less drastic in the viral activities (metatranscriptomics), where Phycodnaviridae family, represented almost ∼50% of the total activity, followed by Marine RNA PAL viruses, which increased up to ∼38% of total RNA viral reads. Meanwhile, the Myoviridae family showed a reduced activity (∼10% of the total viral RNA), in comparison to the L-C sample, consistent with the decrease in DNA abundance.

### Genome Identification and Characterization of Major Viral Groups

Due to the dramatic differences found in the viral communities occurring between L-C and H-C contrasting summer time samples, the major viral groups identified in this study were analyzed more deeply at a genomic level. One dominant complete genome and several partial genomes, belonging to the Myoviridae and Phycodnaviridae families, respectively, were retrieved when both L-C and H-C metagenomes were co-assembled. Also, the complete genomes from a PAL E4 and Pal 156 ssRNA virus were recovered from both individual metatranscriptomic assembly.

**Figure 2:**
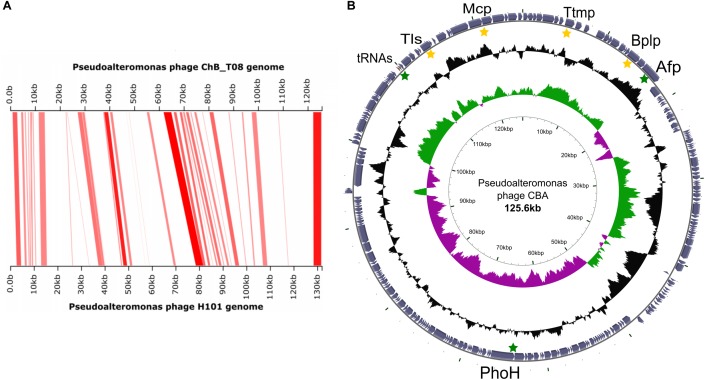
Genomic characteristics of Pseudoalteromonas phage CBA (PpCBA). **(A)** Genomic comparison: Nucleotide comparison of the novel genome of PpCBA, with their closest homologous from the database Pseudoalteromonas phage H101, darker to clearer zones represents highly to less conserved regions while non-connected correspond to divergent regions. **(B)** Circular representation of the PpCBA genome: the different rings represent (from inner to outer), GC skew, deviation from average GC content and predicted proteins. Yellow stars correspond to viral core proteins: Tail tape measure protein (Ttmp), Base plate-like protein (BplP), Terminase large subunit (Tls) and Major capsid protein (Mcp), while green marks indicate relevant accessory metabolic genes, such as the Anti-freezing protein domain (Afp) or tRNA.

Most of the reads associated with the complete Myovirus genome were recruited on two separated contigs with an overlapping region unified on a single sequence of 125.6 kb, named Pp_CBA virus (Pseudoalteromonas phage Chile Bay, Antarctica). The nucleotide alignment to the NAV database showed general similarity in the genome organization between Pp_CBA and the Pseudoalteromonas phage H101 (PpH101), also indicating a nucleotide homology of 24% (Figure 2A). Protein prediction for this new viral genome detected 224 CDS (Supplementary Table 3). Among these CDS, several viral signatures genes were retrieved such as the MCP, the tail tape measure protein (Ttmp), and the base plate-like protein (BplP) (Figure 2B). The Pp_CBA genome exhibited a GC bipartite distribution, where all the structural and hallmark proteins identified were encoded in high GC content regions. Conversely, several metabolic accessory genes were found in low GC content regions, such as the phosphorous transporter phoH, two tRNAs, and an antifreeze protein domain.

**Figure 3:**
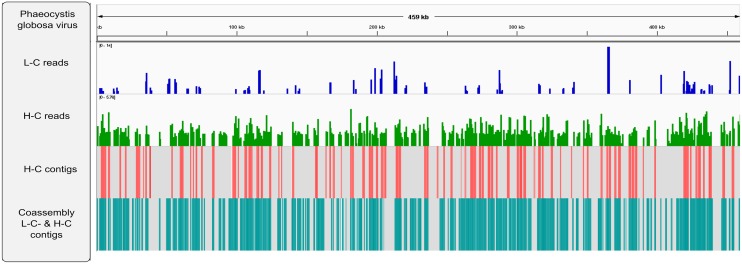
Novel Phycodnaviridae antarctica (PaV) genome recruitment. Alignment of reads and assembled contigs from both metagenomes, as well as contigs from the co-assembly, was performed against the *Phaeocystis globosa* virus 16T (PgV) genome. The blue and green bars indicate the number of reads recruited per region on a logarithmic scale. Red and gray heat maps show the regions that include the PgV genome, with contigs retrieved from the assemblies. The L-C contig recruitment map is not included as no significative contigs were recruited from the assembly.

On the other hand, the dominant viral group identified as Phycodnavirus in the H-C period was represented by multiple contigs in the assemblies being collectively referred to as PaV (Phycodnaviridae Antarctica virus). The PaV contigs aligned with the *Phaeocystis globosa* virus 16T (PgV) genome, with an average nucleotide similarity of 95%. Read recruitment for each metagenome, using the PgV genome as a reference, indicated a greater coverage in the H-C than in the L-C sample (Figure 3). Contig recruitment against PgV recovered 0, 100 and 550 contigs for L-C, H-C, and L-C H-C co-assembly, respectively, giving a total draft genome of 135 kb from both samples. The size of the contigs retrieved enabled the prediction of several partial proteins with a known function, such as DNA polymerase and a MCP (Supplementary Table 4).

For the detected ssRNA viruses, metatranscriptomics reads were aligned against the available RNA genomes on the RefSeq database. They were identified as known members of the Picornaviridae family, with an average nucleotide identity of 99.9%. Metatranscriptomes assembled separately, leading to the recovery of the complete genome of Pal 156 (7.9 kb) in both samples, while the complete genome of PAL E4 (8.19 kb) was only recovered in the H-C sample.

### Phylogenetic Reconstructions of Newly Characterized Viral Genomes

**Figure 4:**
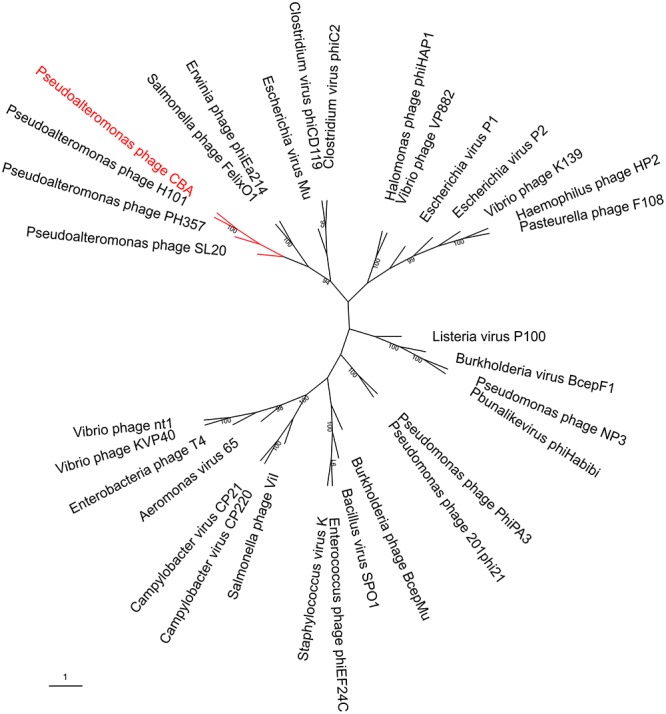
Phylogenetic clustering of PpCBA within the different groups of Myoviridae family. Maximum likelihood approximation was used for the phylogenetic reconstruction analysis. Representative sequences of the major capsid protein of main subfamilies and genus from the Myoviridae family were included, as well as previously unclassified Pseudoalteromonas-infecting phages sequences.

Phylogenetic analysis of Pp_CBA was performed based on Mcp protein sequences from several subfamilies and genera within the Myoviridae family (Figure 4). The phylogeny supports a relationship between Pp_CBA with the Pseudoalteromonas phage H101 and two other unclassified *Pseudoalteromonas* infecting phages (SL20 and PH357), forming a monophyletic clade separated from the sister genus of Felix01 viruses, which mainly infect Enterobacteria. Furthermore, multimarker analyses with VirFam assigned the Pp_CBA virus to the Myoviridae Type1, Cluster 7, closely related to the FelixO1 (Supplementary Figure 2), as was also observed through Mcp protein phylogenetic reconstruction (Figure 4).

A genomic comparison between members of the FelixO1 genus and Pp_CBA revealed variations in the average genome length ∼86 vs. ∼127 kb and the number of proteins encoded (∼131 vs. ∼220, respectively). Alignment of the Pp_CBA genome with members from the FelixO1-like virus family indicated no resemblance between the genome structure between these two clades (Supplementary Figure 3). However, *Pseudoalteromonas* like viruses do share similar genomic organization, with a high syntenic degree between different coding regions, separating these unclassified *Pseudoalteromonas* phages from those of the FelixO1 genus.

**Figure 5:**
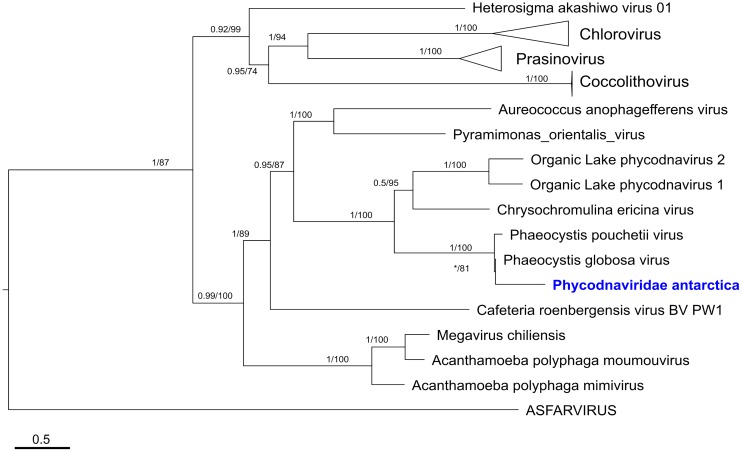
Determination of phylogenetic relations of Phycodnaviridae antarctica. Alignment of DNA PolB protein sequences from Phycodnaviridae and members of the proposed order Megavirales was carried out. Full sequences, as well as partial inferred protein from environmental samples, were included in the analysis. Maximum likelihood and Bayesian approximation consensus tree is represented. The tree was rooted with sequences from the Asfarviridae family. Polytomies obtained by Bayesian approximation are represented by a (^∗^) symbol.

For the Phycodnaviridae draft genome, a phylogenetic reconstruction was performed using the DNA polymerase B marker gene (Figure 5), confirming a relationship between PaV and other giant viruses, as well as with the *Phaeocystis* infecting viruses. All the identified PaV sequences from metagenomic samples from Chile Bay form a well-defined cluster (bootstrap value = 100 and Bayesian value = 1) with Prymnesiovirus sequences, as part of the extended Mimivirus group. Particularly, a monophyletic clade is formed with viruses that infect seawater haptophytes such as the *P. globosa* and *Phaeocystis pouchetii* viruses.

Bayesian inference and maximum likelihood phylogenetic analyses of pol B gene revealed two major branches within the Megavirales order. With one branch of well-defined Phycodnavirus, such as Coccolithovirus, Chlorovirus and Prasinovirus, and another branch including members of the proposed extended Mimivirus group, which include Mimiviruses, Organic lake Phycodnaviruses (OLPv) and Prymnesiovirus.

### Effect of Environmental Factors on Potential Host Abundance and Composition

**Figure 6:**
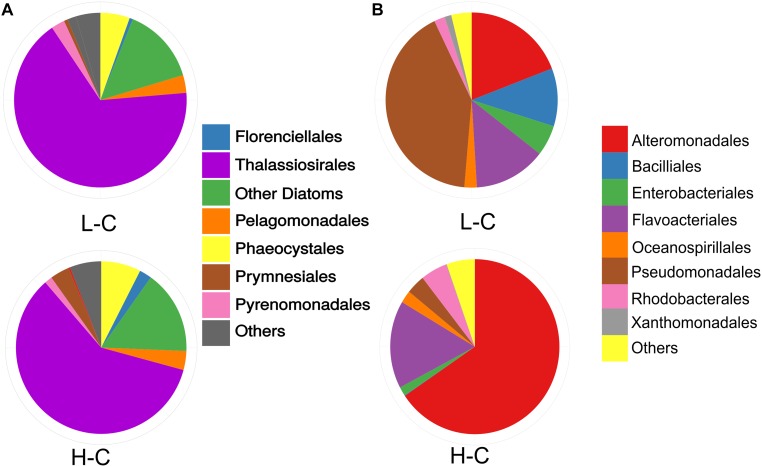
Cellular populations in Chile Bay during a summer phytoplankton bloom in 2014. **(A)** Composition of the eukaryotic phytoplankton community. Relative abundances of the major eukaryotic phytoplankton groups as a percentage of the chloroplastidial 16S rRNA, of 0.22 and 8 μm fractions, obtained by 16S tag sequencing and assigned using the Phytoref database. **(B)** Relative composition of the bacterial community. The relative abundances of the major groups were calculated as a percentage of assigned sequences to particular taxa using all 16S rRNA sequences retrieved from each metagenomic sample. Taxonomy is shown at the level of bacterial orders, taxa with abundance <1% are grouped into the “others” category.

Our analysis, as well as in previous studies (Alcamán-Arias et al., 2018; Fuentes et al., 2018), showed a slight change in the relative abundances and composition of the prokaryotic and eukaryotic communities observed at L-C and H-C samples. Eukaryotic phytoplankton from the L-C sample was dominated by diatoms (Phylum Bacillariophyta; ∼77%) of the orders Thalassiosirales (∼67%) and Bacillariales (∼10%), with the haptophytes (order Phaeocystales; 5%), and the cryptophytes (Pyrenomonadales; ∼3%), representing the second and third major taxa. In the H-C sample, also diatoms (Bacillariophyta; with a relative abundance of up to ∼80%) were the dominant taxon within the eukaryotic community, but the haptophytes increased slightly to ∼11% in comparison with the L-C sample (Figure 6A). In the L-C sample, the relative abundance of cryptophytes decreased by ∼2%, as did other phytoplankton groups that were almost undetectable (less than 1%).

Conversely, the prokaryotic community was dominated by Gammaproteobacteria, in both summertime samples (Figure 6B), representing ∼71% in L-C and 76% in H-C. However, each sample time was dominated by a distinct order. Pseudomonadales was the predominant group (∼42%) in the L-C sample, followed by Flavobacteriales (Bacteroidetes; ∼14%) and Bacillales (Firmicutes; ∼11%). Meanwhile, Alteromonadales dominated (Class Gammaproteobacteria; ∼65%) the H-C sample, followed by the Flavobacteria (16%) and Rhodobacterales (Class Alphaproteobacteria; ∼5%).

Environmental conditions, such as temperature and salinity [increasing from –0.1 to 0.3°C and from ∼33.2 to ∼33.86, respectively (Supplementary Table 4)] show minor variations between L-C and the H-C samples in Chile Bay (Fuentes et al., 2018). The concentration of oxygen and Chl-*a* showed an increase between both samples, going from 148.8% and ∼0.3 mg m^-3^ to 190% and ∼2.53 mg m^-3^, respectively. On the other hand, nitrate and phosphate concentrations slightly decreased, from 24.61 to 2.02 μmol L^-1^ in the L-C sample to 20.16 and 1.59 μmol L^-1^ in the H-C sample. The correlation coefficients calculated from the prokaryotic and eukaryotic composition and the environmental data showed a negative correlation between total Chl-*a* and inorganic nutrients, such as phosphate and nitrate (Supplementary Figure 4). In addition, a positive relationship was observed between the dominant Thalassiosirales order with the bacterial orders Flavobacteriales, Alteromonadales, and Oceanospirillales. With respect to the main viral groups (i.e., Phycodnavirus and RNA Pal viruses), infecting eukaryotes they show a positive correlation with total Chl-*a*, but a negative correlation with the relative abundance of Thalassiosirales, while the dominant bacterioplankton viruses, Myovirus and Inovirus, correlate negatively with the increase in phytoplankton production.

## Discussion

Studying viral ecology in the SO is an issue of priority, considering the importance of this productive ecosystem on a global scale (Evans et al., 2009; Deppeler and Davidson, 2017) and the consequences of lytic infections on nutrient availability through the food webs. Previous studies in Antarctic waters have demonstrated that viral abundance is directly correlated with phytoplankton (measured as Chl-*a* concentration) and bacterial abundances (Evans et al., 2017), where viral infections have a greater influence than grazers on prokaryotic mortality rates, playing a key role in the carbon export to the deep waters (Vaqué et al., 2017). The present multi-omics study identifies some of the major active viruses in Antarctic waters during a phytoplankton bloom in the late summer of 2014 in Chile Bay (WAP). Revealing the most likely hosts, elucidating changes in viral composition and activity between two contrasting stages of the bloom development.

### Identification of Dominant New Viruses Associated With Bacterial Communities

The VBR in the SO has already been identified as remarkably low (0.7 to 6) in comparison to the standard VBR globally observed in the oceans (1 to 50) (Maranger et al., 1994; Wommack and Colwell, 2000). Viral activity together with variations in the abundance of bacteria and phytoplankton has been suggested as a cause of the lower VBR ratio (Evans et al., 2017). Nevertheless, viruses are the major cause of bacterial mortality in the SO, more so than bacterivore activity (Guixa-Boixereu et al., 2002; Evans and Brussaard, 2012; Vaqué et al., 2017). Furthermore, recent time-series studies on the WAP have demonstrated a marked seasonality in virioplankton abundance. Higher abundances are observed in spring and summer months, driven by the increase in bacterioplankton abundance, which in turn is determined by nutrient availability generated by phytoplankton (Evans et al., 2017). Thus, the changes in relative viral abundances observed in this study (measured as total viral reads percent) between L-C and H-C samples in Chile Bay are likely to be due to variations in carbon availability, supplied by phytoplankton, during these two coastal-site contrasting conditions.

In Chile Bay, the phage community associated with bacterioplankton was dominated by a novel *Pseudoalteromonas* virus (Pp_CBA) (Caudovirales). The full Pp_CBA genome retrieved in this study exhibited a high nucleotide identity (24%) and a similar genome organization to Pseudoalteromonas phage H101, isolated from the Yellow Sea, China. Furthermore, despite the geographical distance, ∼50% of the genes are shared between both phages (∼68.7 average similarity at protein level; across 108 of 221 genes with Pp_H101 predicted proteins), suggesting an evolutionary relationship in these cosmopolitan phage lineages (Figure 2B).

Given the phylogenetical relationship, genome homology and structural similarity (Supplementary Figure 3) between Pp_CBA and Gammaproteobacteria-infecting viruses, such as Pseudoalteromonas Phage H101 (PpH101), we propose the *Pseudoalteromonas* genus to be the host of Pp_CBA.

Remarkably, it was also demonstrated that Pp_CBA is not a member of one of the most common and abundant subfamilies of the previously described Myovirus, such as the Peduovirinae (P2), Tevenvirinae (T4), or Spounavirinae (SPO1) (Lavigne et al., 2009). Instead, it is more closely related to the newly described subfamily Ounavirinae (Adriaenssens et al., 2017). The comparison between sequence relatedness and genomic features (i.e., sequence identity, genomic structure, proteins encoded), as shown in Supplementary Figure 2, also suggest, that Felix O1 viruses are not part of the group that includes the *Pseudoalteromonas* phages Pp_CBA, PpH101, PpSL20, and PpPH357. In line with the proposed guidelines for metagenomic virus identification (Adriaenssens and Brister, 2017; Simmonds et al., 2017; Roux et al., 2019), it is concluded that these viruses belong to an undescribed genus within the Ounavirinae subfamily. This new lineage may play an important role in regulating the most abundant heterotrophic bacteria (*Pseudoalteromonas*) identified in Chile Bay, which is also one of the most abundant and widely distributed marine bacterial genus (Skovhus et al., 2007). Thus, as previously suggested, this new lineage may impact on a global biogeochemical scale (Wichels et al., 2002).

### Interaction of Pp_CBA Virus With Its Host and Changes in the Viral Life Cycle

Due to the increased abundance of the order Caudovirales, and in order to gain a better understanding of host interaction and dynamics, putative adaptation strategies able to respond to different environmental conditions in viral genomes were explored, including uncharacterized and non-structural proteins from low GC-content regions (more prone to vary) (Lassalle et al., 2015). These regions incorporate metabolic accessory genes to ensure host survival during viral replication (Roux et al., 2016). For example, an anti-freezing protein with a collagen binding-like domain has been identified in the low GC region of the Pp_CBA genome. This protein is reported to inhibit ice recrystallization in frozen solutions and maintain intracellular colloidal conditions (Knight and Duman, 1986). Another example is the presence of a *pho*H-like gene, a phosphorous transporter that is expressed during nutrient starvation, and described as a common phage signature (Goldsmith et al., 2015). In addition, other capacities have been described for Arctic isolated *Pseudoalteromonas* phages, providing advantageous host characteristics, such as enhanced motility and chemotaxis (Yu et al., 2015). Thus, the presence of metabolic accessory genes in a low-temperature environment is suggested to be a strategy that ensures a successful life-cycle strategy in this extreme environment.

In Chile Bay, the increase in *Pseudoalteromonas* abundance and a decrease in viral activity of Pp_CBA in the H-C sample (Figure 1, 6B) suggests viral variations contrary to expectations set by the “Killing the Winner” hypothesis (Thingstad and Lignell, 1997; Winter et al., 2010). This behavior, based on metabolic accessory genes and variations in DNA/RNA, may be a consequence of the variation from a lytic to a lysogenic strategy. This change is facilitated through the presence of tRNA, identified as a prophage integration site (Williams, 2002), as well as several recombinase enzymes codified on the PpCBA genome, or a pseudolysogeny state, which remains a circular extrachromosomal element, as described for several gammaproteobacterial virus–hosts interactions (Mobeus, 1997; Rippt and Miller, 1998). This is similar to the behavior described as the Piggyback-the-Winner model, where the temperate viral strategy becomes increasingly important in ecosystems with high microbial densities (Knowles et al., 2016). The latter is translated into a negative association between host abundance and the virus-to-host ratio, reducing phage lysis at higher host densities (Knowles et al., 2016; Coutinho et al., 2017).

### Influence of Phytoplankton-Infecting DNA and RNA Viruses on the Productivity of the Coastal Waters of the Southern Ocean

In Antarctic waters, the majority of the currently described DNA viruses are within the order Caudovirales, composing up to 80% of the viral community (Brum et al., 2016). As is the case in Chile Bay during the Antarctic L-C condition (Figure 1). However, under high Chl-*a* conditions (still into the range for Antarctic coastal waters) a change in the dominant groups of the viral community was observed. Here, the Prymnesiovirus PaV related sequences, that form part of the extended Mimivirus group, are identified to be the most abundant community members. These results suggest that the structure and composition of the viral community in Chile Bay vary throughout the austral summer, can change from a bacterial-viruses dominated community to a eukaryotic-viruses dominated one, during periods of high productivity (high Chl-*a* concentration). The situation reverses as phytoplankton bloom collapses, returning to a bacteriophage-dominated community. The increase in viral DNA from Prymnesiovirus PaV is associated with an increase in viral particles, likely due to viral lysis of the Prymnesiales host. This increases activity from 12.8 to 25.8% of the total photosynthetic community in the L-C and H-C period, respectively (Alcamán-Arias et al., 2018). The high activity of the Phycodnaviridae family observed on the L-C and H-C samples suggest a persistent and active haptophytes infection.

Phylogenetic analyses of Pol B explore the relationships between the Nucleocytoplasmic Large DNA Viruses (NCLDV) (Koonin et al., 2015). Results indicate PaV is within the Primnesiovirus group, forming part of the previously termed “extended Mimiviridae family” (Moniruzzaman et al., 2016). This group comprises two main clades, the heterotrophic-host infecting virus, such as *Acanthamoeba polyphaga* virus and *Cafeteria roenbergensis* virus; and the photosynthetic-host infecting virus, including the *Aureococcus anophagefferens* virus, *Pyramimonas orientalis* virus and the viruses infecting Prymnesiales (Moniruzzaman et al., 2016). The PaV sequences form a cluster with *P. globosa* 16T and *P. pouchetii* viruses. Thus far, viruses infecting *Phaeocystis* spp. are species-specific (Jacobsen et al., 1996), with hosts (*P. globosa* and *P. pouchetii*) presenting different biogeography’s and absent from Antarctic waters (Schoemann et al., 2005; Santinia et al., 2013). Thus, *P. antarctica* is suggested being the PaV putative host, being the dominant and only *Phaeocystis* blooming species in the SO (Arrigo, 1999; Schoemann et al., 2005). To date, no viruses have been previously reported for this species and further infection assays are necessary to confirm this relationship in Antarctic waters.

This virus may have major impacts on SO biogeochemical cycles. In addition to organic primary production, *P. antarctica* is also a major producer of dimethylsulfoniopropionate (DMSP), a sulfur compound (Gibson et al., 1990; Asher et al., 2017). This compound is the precursor to dimethyl sulfide (DMS), the major oceanic source of sulfur volatile compounds (Curran and Jones, 2000). DMS is considered to be a cloud condensation nuclei agent, influencing the albedo (and thus the light availability) for primary production post bloom, and subsequently affecting global climate change through augmenting the greenhouse effect (Iversen and Seland, 2002; Crutzen and Andreae, 2006). Furthermore, DMSP is used as a carbon source by many marine bacteria, such as Rhodobacterales, which increased (from ∼2 to 5%) in Chile bay during the H-C period. DMSP availability due to viral lysis potentially has a powerful influence on the microbial community composition during *P. antarctica* blooms (Kiene and Linn, 2000). Under this scenario, the novel *P. antarctica* virus (PaV) may play an important ecological and climatic role through changes in the carbon and sulfur biogeochemical cycles. For example, modulating the carbon drag and primary production, as well as affecting the SO albedo. In order to gain a better understanding of the magnitude of these events, further investigation is necessary for viral effects and DMS emission.

Conversely, RNA viral sequences related to the marine RNA virus PAL E4 and PAL 156 were detected in Chile Bay, presenting increased abundance in the H-C sample (Figure 1). These RNA viral members have no DNA stage, thus the metatranscriptomic approach was unable to distinguish genomic RNA from activity (expressed transcripts). Nevertheless, considering the cellular fraction between 8 and 0.22 μm was sequenced, it is assumed that the majority of recovered viral sequences are from attached particles, or active infections replicating inside hosts. RNA and DNA viruses have been reported to infect pennate and centric blooming and non-blooming diatoms, and they play a major role in oceanic phytoplankton bloom decay (Bratbak et al., 1990; Nagasaki, 2008). Recently, the overall increase in the activity and/or presence of PAL E4 and PAL156 viruses, both belonging to Picornavirales group, has been reported near to Palmer station (WAP), during a spring-summer transition along with recurrent diatom blooms (Miranda et al., 2016). Phylogenetic analyses of Antarctic PAL156 sequences showed that they were closely related to the Bacillarnavirus family, which infect centric diatoms. In Chile Bay, Thalassiosirales (centric diatoms) are dominating along with other types of diatoms (Figure 6A), indicating that these groups are potential Bacillarnavirus hosts, as already reported in other environments (Tomaru et al., 2013). Moreover, PAL E4 shares the bicistronic genome from the Picornavirus group; however, it belongs to a distinct clade with no known host (Miranda et al., 2016), which may be infecting another eukaryote in this bloom. RNA viruses that increase in abundances in parallel to blooming diatoms may play a key role in the regulation of the bloom intensity and duration, and therefore influence the primary productivity in Chile Bay and other WAP regions.

## Concluding Remarks

To our knowledge, this is the first multi-omic approach study where viral communities are identified and compared under different conditions of low and high chlorophyll in the SO. In addition, host interactions were identified from the most abundant and active viral communities in WAP, along with changes in productivity (according to Chl-*a* measurements) and the dominance of eukaryotic phytoplankton and bacterial communities. Finally, novel genomes belonging to the most abundant viruses were recovered, while genomic analyses revealed a new genus within the Myoviridae group, and the first virus (Phycodnavirus) described that can infect *P. antarctica*, an important primary producer in the Antarctic marine food webs.

Although our sampling effort was limited to comparing two samples of contrasting scenarios of different productivity (based on chlorophyll concentration), both samples are representative of the changes in microbial communities of the coastal waters in the WAP during the summer season. The community structure of phytoplankton and bacterioplankton, as well as the concentrations of Chl-*a* (up to 2.53 mg/m^3^) observed in the samples of the present study, are within the ranges previously observed in other nearby locations in the WAP such as Palmer. In addition, and as in Palmer station (Luria et al., 2016; Mangoni et al., 2017) the microbial communities of Chile Bay during phytoplankton blooms were dominated by Alfa and Gammaproteobacteria, as well as Flavobacteria. This suggests that the changes observed in the viral communities in our study can be used as a model to understand the behavior of virioplankton in the coastal waters along the WAP, highlighting their potential role as regulators of important phytoplankton and bacterioplankton components in the coastal Antarctic waters. The latter is also supported by our phylogenetic analyses of the two new genomes discovered here, which explain the existence of closely related viruses in other marine environments that are known to play an important role in these food webs.

## Author Contributions

TA-S and SG-L contributed to the experimental design, data analyses, and manuscript redaction. JA contributed actively with the interpretation of the results and contributed to the final version of the manuscript. BD contributed to the design and implementation of the research and supervised the development and findings of this work. All authors discussed the results and contributed to the final manuscript.

## Conflict of Interest Statement

The authors declare that the research was conducted in the absence of any commercial or financial relationships that could be construed as a potential conflict of interest.

## References

[B1] Adriaenssens E. M. Brister J. R. (2017). How to name and classify your phage: an informal guide. *Viruses* 9 1–9. 10.3390/v9040070 28368359 PMC5408676

[B2] Adriaenssens E. M. Krupovic M. Knezevic P. Ackermann H. W. Barylski J. Brister J. R. (2017). Taxonomy of prokaryotic viruses: 2016 update from the ICTV bacterial and archaeal viruses subcommittee. *Arch. Virol.* 162 1153–1157. 10.1007/s00705-016-3173-4 28040838

[B3] Alcamán-Arias M. Farias L. Verdugo J. Alarcon-Schumacher T. Diez B. (2018). Microbial activity during a coastal phytoplankton bloom on the western antarctic peninsula in late summer. *FEMS Microbiol. Lett.* 365 1–51. 29788084 10.1093/femsle/fny090

[B4] Andrews S. (2010). *FastQC**: a Quality Control Tool for High Throughput Sequence Data* Available at: https://www.bioinformatics.babraham.ac.uk/projects/fastqc/

[B5] Armstrong F. A. J. Stearns C. R. Strickland J. D. H. (1967). The measurement of upwelling and subsequent biological process by means of the technicon autoanalyzer^®^ and associated equipment. *Deep. Res. Oceanogr. Abstr.* 14 381–389. 10.1016/0011-7471(67)90082-4

[B6] Arrigo K. R. (1999). Phytoplankton community structure and the drawdown of nutrients and CO2 in the Southern Ocean. *Science* 283 365–367. 10.1126/science.283.5400.365 9888847

[B7] Asher E. C. Dacey J. W. H. Stukel M. Long M. C. Tortell P. D. (2017). Processes driving seasonal variability in DMS, DMSP, and DMSO concentrations and turnover in coastal Antarctic waters. *Limnol. Oceanogr.* 62 104–124. 10.1002/lno.10379

[B8] Bankevich A. Nurk S. Antipov D. Gurevich A. A. Dvorkin M. Kulikov A. S. (2012). SPAdes: a new genome assembly algorithm and its applications to single-cell sequencing. *J. Comput. Biol.* 19 455–477. 10.1089/cmb.2012.0021 22506599 PMC3342519

[B9] Bergh Ø BØrsheim K. Y. Bratbak G. Heldal M. (1989). High abundance of viruses found in aquatic environments. *Nature* 340 467–468. 10.1038/340467a0 2755508

[B10] Boisvert S. Raymond F. Godzaridis É Laviolette F. Corbeil J. (2012). Ray Meta: scalable de novo metagenome assembly and profiling. *Genome Biol.* 13:R122. 10.1186/gb-2012-13-12-r122 23259615 PMC4056372

[B11] Bratbak G. Heldal M. Norland S. Thingstad T. F. (1990). Partners in spring bloom microbial trophodynamics. *Appl. Environ. Microbiol.* 56 1400–1405. 16348190 10.1128/aem.56.5.1400-1405.1990 PMC184418

[B12] Brum J. R. Hurwitz B. L. Schofield O. Ducklow H. W. Sullivan M. B. (2016). Seasonal time bombs: dominant temperate viruses affect Southern Ocean microbial dynamics. *ISME J.* 10 437–449. 10.1038/ismej.2015.125 26296067 PMC4737935

[B13] Brussaard C. P. D. Short S. M. Frederickson C. M. Suttle C. A. (2004). Isolation and Phylogenetic Analysis of Novel Viruses Infecting the Phytoplankton. *Society* 70 3700–3705. 10.1128/AEM.70.6.3700 15184176 PMC427783

[B14] Bunse C. Pinhassi J. (2017). Marine bacterioplankton seasonal succession dynamics. *Trends Microbiol.* 25 494–505. 10.1016/j.tim.2016.12.013 28108182

[B15] Camacho C. Coulouris G. Avagyan V. Ma N. Papadopoulos J. Bealer K. (2009). BLAST+: architecture and applications. *BMC Bioinformatics* 10:421. 10.1186/1471-2105-10-421 20003500 PMC2803857

[B16] Coutinho F. H. Silveira C. B. Gregoracci G. B. Thompson C. C. Edwards R. A. Brussaard C. P. D. (2017). Marine viruses discovered via metagenomics shed light on viral strategies throughout the oceans. *Nat. Commun.* 8 1–12. 10.1038/ncomms15955 28677677 PMC5504273

[B17] Crutzen P. J. Andreae M. O. (2006). Biomass burning in the tropics: impact on atmospheric chemistry and biogeochemical cycles. *Science* 250 1669–1678. 10.1126/science.250.4988.1669 17734705

[B18] Curran M. A. J. Jones B. (2000). Dimethyl sulfide in the Southern Ocean: seasonality and flux. *J. Geophys. Res. Atmos.* 105 451–459.

[B19] Darling A. C. E. Mau B. Blattner F. R. Perna N. T. (2004). Mauve: multiple alignment of conserved genomic sequence with rearrangements mauve: multiple alignment of conserved genomic sequence with rearrangements. *Genome Res.* 14 1394–1403. 10.1101/gr.2289704 15231754 PMC442156

[B20] Decelle J. Romac S. Stern R. F. Bendif E. M. Zingone A. Audic S. (2015). PhytoREF: a reference database of the plastidial 16S rRNA gene of photosynthetic eukaryotes with curated taxonomy. *Mol. Ecol. Resour.* 15 1435–1445. 10.1111/1755-0998.12401 25740460

[B21] Delcher A. L. Phillippy A. Carlton J. Salzberg S. L. (2002). Fast algorithms for large-scale genome alignment and comparison. *Nucleic Acids Res.* 30 2478–2483. 10.1093/nar/30.11.2478 12034836 PMC117189

[B22] Delmont T. O. Hammar K. M. Ducklow H. W. Yager P. L. Post A. F. (2014). Phaeocystis antarctica blooms strongly influence bacterial community structures in the Amundsen sea polynya. *Front. Microbiol.* 5:646. 10.3389/fmicb.2014.00646 25566197 PMC4271704

[B23] Deppeler S. L. Davidson A. T. (2017). Southern ocean phytoplankton in a changing climate. *Front. Mar. Sci.* 4:40. 10.3389/fmars.2017.00040 27236210

[B24] Díez B. Pedrós-Alió C. Massana R. (2001). Study of genetic diversity of eukaryotic picoplankton in different oceanic regions by small-subunit rRNA gene cloning and sequencing. *Appl. Environ. Microbiol.* 67 2932–2941. 10.1128/AEM.67.7.2932-2941.2001 11425705 PMC92964

[B25] Ducklow H. W. Schofield O. Vernet M. Stammerjohn S. Erickson M. (2012). Multiscale control of bacterial production by phytoplankton dynamics and sea ice along the western Antarctic peninsula: a regional and decadal investigation. *J. Mar. Syst.* 98–99, 26–39. 10.1016/j.jmarsys.2012.03.003

[B26] Evans C. Brandsma J. Pond D. W. Venables H. J. Meredith M. P. Witte H. J. (2017). Drivers of interannual variability in virioplankton abundance at the coastal western Antarctic peninsula and the potential effects of climate change. *Environ. Microbiol.* 19 740–755. 10.1111/1462-2920.13627 27902869

[B27] Evans C. Brussaard C. P. D. (2012). Regional variation in lytic and lysogenic viral infection in the Southern ocean and its contribution to biogeochemical cycling. *Appl. Environ. Microbiol.* 78 6741–6748. 10.1128/AEM.01388-12 22798377 PMC3426681

[B28] Evans C. Pearce I. Brussaard C. P. D. (2009). Viral-mediated lysis of microbes and carbon release in the sub-Antarctic and polar frontal zones of the Australian Southern Ocean. *Environ. Microbiol.* 11 2924–2934. 10.1111/j.1462-2920.2009.02050.x 19758350

[B29] Fuentes S. Arroyo J. I. Rodríguez-Marconi S. Masotti I. Alarcón-Schumacher T. Polz M. F. (2018). Summer phyto- and bacterioplankton communities during low and high productivity scenarios in the Western Antarctic peninsula. *Polar Biol.* 42 159–169. 10.1007/s00300-018-2411-5

[B30] Gibson J. A. E. Garrick R. C. Burton H. R. McTaggart A. R. (1990). Dimethylsulfide and the alga Phaeocystis pouchetii in antarctic coastal waters. *Mar. Biol.* 104 339–346. 10.1007/BF01313276

[B31] Giovannoni S. J. Vergin K. L. (2012). Seasonality in ocean microbial communities. *Science* 335 671–676. 10.1126/science.1198078 22323811

[B32] Goldsmith D. B. Parsons R. J. Beyene D. Salamon P. Breitbart M. (2015). Deep sequencing of the viral phoH gene reveals temporal variation, depth-specific composition, and persistent dominance of the same viral phoH genes in the Sargasso Sea. *PeerJ* 3:e997. 10.7717/peerj.997 26157645 PMC4476143

[B33] Guixa-Boixereu N. Vaqué D. Gasol J. M. Sánchez-Cámara J. Pedrós-Alió C. (2002). Viral distribution and activity in Antarctic waters. *Deep. Res. Part II Top. Stud. Oceanogr.* 49 827–845. 10.1016/S0967-0645(01)00126-6 22798377

[B34] Huson D. H. Beier S. Flade I. Górska A. El-Hadidi M. Mitra S. (2016). MEGAN community edition - interactive exploration and analysis of large-scale microbiome sequencing data. *PLoS Comput. Biol.* 12:e1004957. 10.1371/journal.pcbi.1004957 27327495 PMC4915700

[B35] Hyatt D. Chen G. Locascio P. F. Land M. L. Larimer F. W. Hauser L. J. (2010). Prodigal: prokaryotic gene recognition and translation initiation site identification. *BMC Bioinformatics* 11:119. 10.1186/1471-2105-11-119 20211023 PMC2848648

[B36] Iversen T. Seland Ø (2002). A scheme for process-tagged SO *< inf > 4 < /inf >* and BC aerosols in NCAR CCM3: validation and sensitivity to cloud processes. *J. Geophys. Res. Atmos.* 107:4751 10.1029/2001JD000885

[B37] Jacobsen A. Bratbak G. Heldal M. (1996). Isolation and characterization of a virus infecting *Phaeocystis pouchetii* (Prymnesiophyceae). *J. Phycol.* 32 923–927. 10.1111/j.0022-3646.1996.00923.x

[B38] Jones P. Binns D. Chang H. Y. Fraser M. Li W. McAnulla C. (2014). InterProScan 5: genome-scale protein function classification. *Bioinformatics* 30 1236–1240. 10.1093/bioinformatics/btu031 24451626 PMC3998142

[B39] Katoh K. Standley D. M. (2013). MAFFT multiple sequence alignment software version 7: improvements in performance and usability. *Mol. Biol. Evol.* 30 772–780. 10.1093/molbev/mst010 23329690 PMC3603318

[B40] Kiene R. P. Linn L. J. (2000). The fate of dissolved dimethylsulfoniopropionate (DMSP) in seawater: tracer studies using 35S-DMSP. *Geochim. Cosmochim. Acta* 64 2797–2810. 10.1016/S0016-7037(00)00399-9

[B41] Knight C. A. Duman J. G. (1986). Inhibition of recrystallization of ice by insect thermal hysteresis proteins: a possible cryoprotective role. *Cryobiology* 23 256–262. 10.1016/0011-2240(86)90051-9

[B42] Knowles B. Silveira C. B. Bailey B. A. Barott K. Cantu V. A. Cobian-Guëmes A. G. (2016). Lytic to temperate switching of viral communities. *Nature* 531 466–470. 10.1038/nature17193 26982729

[B43] Koonin E. V. Krupovic M. Yutin N. (2015). Evolution of double-stranded DNA viruses of eukaryotes: from bacteriophages to transposons to giant viruses. *Ann. N. Y. Acad. Sci.* 1341 10–24. 10.1111/nyas.12728 25727355 PMC4405056

[B44] Langmead B. Salzberg S. L. (2012). Fast gapped-read alignment with bowtie 2. *Nat. Methods* 9 357–359. 10.1038/nmeth.1923 22388286 PMC3322381

[B45] Lassalle F. Périan S. Bataillon T. Nesme X. Duret L. Daubin V. (2015). GC-content evolution in bacterial genomes: the biased gene conversion hypothesis expands. *PLoS Genet.* 11:e1004941. 10.1371/journal.pgen.1004941 25659072 PMC4450053

[B46] Lavigne R. Darius P. Summer E. J. Seto D. Mahadevan P. Nilsson A. S. (2009). Classification of Myoviridae bacteriophages using protein sequence similarity. *BMC Microbiol.* 9:224. 10.1186/1471-2180-9-224 19857251 PMC2771037

[B47] Lawrence J. E. Suttle C. A. (2004). Effect of viral infection on sinking rates of Heterosigma akashiwo and its implications for bloom termination. *Aquat. Microb. Ecol.* 37 1–7. 10.3354/ame037001

[B48] Le S. Q. Gascuel O. (2008). An improved general amino acid replacement matrix. *Mol. Biol. Evol.* 25 1307–1320. 10.1093/molbev/msn067 18367465

[B49] Lopes A. Tavares P. Petit M.-A. Guérois R. Zinn-Justin S. (2014). Automated classification of tailed bacteriophages according to their neck organization. *BMC Genomics* 15:1027. 10.1186/1471-2164-15-1027 25428721 PMC4362835

[B50] Luria C. M. Amaral-Zettler L. A. Ducklow H. W. Rich J. J. (2016). Seasonal succession of free-living bacterial communities in coastal waters of the western antarctic peninsula. *Front. Microbiol.* 7:1731. 10.3389/fmicb.2016.01731 27857708 PMC5093341

[B51] Mangoni O. Saggiomo V. Bolinesi F. Margiotta F. Budillon G. Cotroneo Y. (2017). Phytoplankton blooms during austral summer in the Ross Sea, Antarctica: driving factors and trophic implications. *PLoS One* 12:e0176033. 10.1371/journal.pone.0176033 28430813 PMC5400245

[B52] Maranger R. Bird D. F. Karl D. M. (1994). Palmer LTER: spatial distribution of viruses in the Palmer LTER region. *Antarct. J. U.S.* 29 209–211.

[B53] Mendes C. R. B. de Souza M. S. Garcia V. M. T. Leal M. C. Brotas V. Garcia C. A. E. (2012). Dynamics of phytoplankton communities during late summer around the tip of the Antarctic Peninsula. *Deep. Res. Part I Oceanogr. Res. Pap.* 65 1–14. 10.1016/j.dsr.2012.03.002

[B54] Miranda J. A. Culley A. I. Schvarcz C. R. Steward G. F. (2016). RNA viruses as major contributors to Antarctic virioplankton. *Environ. Microbiol.* 18 3714–3727. 10.1111/1462-2920.13291 26950773

[B55] Mobeus K. (1997). Investigations of the marine lysogenic bacterium H24. I. development of pseudolysogeny in nutrient-rich broth culture. *Mar. Ecol. Prog. Ser.* 148 229–240. 10.3354/meps148229

[B56] Moniruzzaman M. Gann E. R. LeCleir G. R. Kang Y. Gobler C. J. Wilhelm S. W. (2016). Diversity and dynamics of algal Megaviridae members during a harmful brown tide caused by the pelagophyte, aureococcus anophagefferens. *FEMS Microbiol. Ecol.* 92:fiw058. 10.1093/femsec/fiw058 26985013

[B57] Morgulis A. Gertz E. M. Schäffer A. A. Agarwala R. (2006). A fast and symmetric DUST implementation to mask low-complexity DNA sequences. *J. Comput. Biol.* 13 1028–1040. 10.1089/cmb.2006.13.1028 16796549

[B58] Nagasaki K. (2008). Dinoflagellates, diatoms, and their viruses. *J. Microbiol.* 46 235–243. 10.1007/s12275-008-0098-y 18604491

[B59] Piquet A. M. T. Bolhuis H. Meredith M. P. Buma A. G. J. (2011). Shifts in coastal Antarctic marine microbial communities during and after melt water-related surface stratification. *FEMS Microbiol. Ecol.* 76 413–427. 10.1111/j.1574-6941.2011.01062.x 21303395

[B60] Quast C. Pruesse E. Yilmaz P. Gerken J. Schweer T. Yarza P. (2013). The SILVA ribosomal RNA gene database project: improved data processing and web-based tools. *Nucleic Acids Res.* 41 590–596. 10.1093/nar/gks1219 23193283 PMC3531112

[B61] Rambaut A. Drummond A. J. Xie D. Baele G. Suchard M. A. (2018). Posterior summarization in bayesian phylogenetics using tracer 1.7. *Syst. Biol.* 67 901–904. 10.1093/sysbio/syy032 29718447 PMC6101584

[B62] Riemann L. Winding A. (2001). Community dynamics of free-living and particle-associated bacterial assemblages during a freshwater phytoplankton bloom. *Microb. Ecol.* 42 274–285. 10.1007/s00248-001-0018-8 12024253

[B63] Rippt S. Miller R. V. (1998). Dynamics of the pseudolysogenic response in slowly growing cells of Pseudomonas aeruginosa. *Microbiology* 144(Pt 8), 2225–2232. 10.1099/00221287-144-8-2225 9720044

[B64] Ronquist F. Teslenko M. van der Mark P. Ayres D. L. Höhna S. Larget B. (2012). MrBayes 3.2: efficient bayesian phylogenetic inference and model choice across a large model space. *Syst. Biol.* 61 539–542. 10.1093/sysbio/sys029 22357727 PMC3329765

[B65] Rooney-Varga J. N. Giewat M. W. Savin M. C. Sood S. Legresley M. Martin J. L. (2005). Links between phytoplankton and bacterial community dynamics in a coastal marine environment. *Microb. Ecol.* 49 163–175. 10.1007/s00248-003-1057-0 15688258

[B66] Roux S. Adriaenssens E. M. Dutilh B. E. Koonin E. V. Kropinski A. M. Krupovic M. (2019). Minimum information about an uncultivated virus genome (MIUVIG). *Nat. Biotechnol.* 37 29–37. 10.1038/nbt.4306 30556814 PMC6871006

[B67] Roux S. Brum J. R. Dutilh B. E. Sunagawa S. Duhaime M. B. Loy A. (2016). Ecogenomics and potential biogeochemical impacts of globally abundant ocean viruses. *Nature* 537 689–693. 10.1038/nature19366 27654921

[B68] Santinia S. Jeudya S. Bartolia J. Poirota O. Lescota M. Abergela C. (2013). Genome of Phaeocystis globosa virus PgV-16T highlights the common ancestry of the largest known DNA viruses infecting eukaryotes. *Proc. Natl. Acad. Sci.* 110 10800–10805. 10.1073/pnas.1303251110 23754393 PMC3696832

[B69] Schmieder R. Edwards R. (2011). Quality control and preprocessing of metagenomic datasets. *Bioinformatics* 27 863–864. 10.1093/bioinformatics/btr026 21278185 PMC3051327

[B70] Schmieder R. Lim Y. W. Edwards R. (2012). Identification and removal of ribosomal RNA sequences from metatranscriptomes. *Bioinformatics* 28 433–435. 10.1093/bioinformatics/btr669 22155869 PMC3268242

[B71] Schoemann V. Becquevort S. Stefels J. Rousseau V. Lancelot C. (2005). Phaeocystis blooms in the global ocean and their controlling mechanisms: a review. *J. Sea Res.* 53 43–66. 10.1016/j.seares.2004.01.008

[B72] Schofield O. Saba G. Coleman K. Carvalho F. Couto N. Ducklow H. (2017). Decadal variability in coastal phytoplankton community composition in a changing West Antarctic Peninsula. *Deep. Res. Part I Oceanogr. Res. Pap.* 124 42–54. 10.1016/j.dsr.2017.04.014

[B73] Simmonds P. Adams M. J. Benk M. Breitbart M. Brister J. R. Carstens E. B. (2017). Consensus statement: virus taxonomy in the age of metagenomics. *Nat. Rev. Microbiol.* 15 161–168. 10.1038/nrmicro.2016.177 28134265

[B74] Skovhus T. L. Holmström C. Kjelleberg S. Dahllöf I. (2007). Molecular investigation of the distribution, abundance and diversity of the genus *Pseudoalteromonas* in marine samples. *FEMS Microbiol. Ecol.* 61 348–361. 10.1111/j.1574-6941.2007.00339.x 17573938

[B75] Stothard P. Wishart D. S. (2005). Circular genome visualization and exploration using CGView. *Bioinformatics* 21 537–539. 10.1093/bioinformatics/bti054 15479716

[B76] Strickland J. Parsons T. (1972). *A Practical Handbook of Seawater Analysis.* Ottawa, ON: Fisheries Research Board of Canada.

[B77] Suttle C. A. (2005). Viruses in the sea. *Nature* 437 356–361. 10.1038/nature04160 16163346

[B78] Takahashi T. Sutherland S. C. Colm S. Poisson A. Metzl N. Tilbrook B. (2002). Global sea–air CO2 flux based on climatological surface ocean pCO2, and seasonal biological and temperature effects. *Deep Sea Res. Part II Top. Stud. Oceanogr.* 49 1601–1622. 10.1016/s0967-0645(02)00003-6

[B79] Tatusova T. DiCuccio M. Badretdin A. Chetvernin V. Nawrocki E. P. Zaslavsky L. (2016). NCBI prokaryotic genome annotation pipeline. *Nucleic Acids Res.* 44 6614–6624. 10.1093/nar/gkw569 27342282 PMC5001611

[B80] Thingstad T. F. Lignell R. (1997). Theoretical models for the control of bacterial growth rate, abundance, diversity and carbon demand. *Aquat. Microb. Ecol.* 13 19–27. 10.3354/ame013019

[B81] Thomsen J. Johnson K. S. Petty R. L. (1983). Determination of silicate in brackish or seawater by flow injection analysis. *Anal. Chem.* 55 2378–2382. 10.1021/ac00264a039

[B82] Tomaru Y. Toyoda K. Kimura K. Takao Y. Sakurada K. Nakayama N. (2013). Isolation and characterization of a single-stranded RNA virus that infects the marine planktonic diatom *Chaetoceros* sp. (SS08-C03). *Phycol. Res.* 61 27–36. 10.1111/j.1440-1835.2012.00670.x

[B83] Trifinopoulos J. Nguyen L. T. von Haeseler A. Minh B. Q. (2016). W-IQ-TREE: a fast online phylogenetic tool for maximum likelihood analysis. *Nucleic Acids Res.* 44 W232–W235. 10.1093/nar/gkw256 27084950 PMC4987875

[B84] Vaqué D. Boras J. A. Torrent-Llagostera F. Agustí S. Arrieta J. M. Lara E. (2017). Viruses and protists induced-mortality of prokaryotes around the antarctic peninsula during the Austral summer. *Front. Microbiol.* 8:241. 10.3389/fmicb.2017.00241 28303119 PMC5332362

[B85] Vernet M. Martinson D. Iannuzzi R. Stammerjohn S. Kozlowski W. Sines K. (2008). Primary production within the sea-ice zone west of the Antarctic Peninsula: i-sea ice, summer mixed layer, and irradiance. *Deep. Res. Part II Top. Stud. Oceanogr.* 55 2068–2085. 10.1016/j.dsr2.2008.05.021

[B86] Wichels A. Gerdts G. Schütt C. (2002). *Pseudoalteromonas* spp. phages, a significant group of marine bacteriophages in the North Sea. *Aquat. Microb. Ecol.* 27 233–239. 10.3354/ame027233

[B87] Wickham H. (2017). ggplot2 – elegant graphics for data analysis (2nd Edition). *J. Stat. Softw.* 77 2–5. 10.18637/jss.v077.b02

[B88] Williams K. P. (2002). Integration sites for genetic elements in prokaryotic tRNA and tmRNA genes: sublocation preference of integrase subfamilies. *Nucleic Acids Res.* 30 866–875. 10.1093/nar/30.4.866 11842097 PMC100330

[B89] Winter C. Bouvier T. Weinbauer M. G. Thingstad T. F. (2010). Trade-offs between competition and defense specialists among unicellular planktonic organisms: the “killing the winner”. hypothesis revisited. *Microbiol. Mol. Biol. Rev.* 74 42–57. 10.1128/MMBR.00034-09 20197498 PMC2832346

[B90] Wintersinger J. A. Wasmuth J. D. (2014). Kablammo: an interactive, web-based BLAST results visualizer Jeff. *Brief. Bioinform.* 15 484–503. 10.1093/bib/bbt009 25481007

[B91] Wommack K. E. Colwell R. R. (2000). Virioplankton: viruses in aquatic ecosystems. *Microbiol. Mol. Biol. Rev.* 64 69–114. 10.1128/MMBR.64.1.69-114.2000 10704475 PMC98987

[B92] Yu Z. C. Chen X. L. Shen Q. T. Zhao D. L. Tang B. L. Su H. N. (2015). Filamentous phages prevalent in Pseudoalteromonas spp. Confer properties advantageous to host survival in Arctic sea ice. *ISME J.* 9 871–881. 10.1038/ismej.2014.185 25303713 PMC4817708

[B93] Yutin N. Wolf Y. I. Koonin E. V. (2015). Origin of giant viruses from smaller DNA viruses not from a fourth domain of cellular life. *Virology* 466-467 38–52. 25042053 10.1016/j.virol.2014.06.032 PMC4325995

