# Supplementary material for: Elucidating Viral Communities During a Phytoplankton Bloom on the West Antarctic Peninsula
Source: Front Microbiol. 2019 May 14;10:1014. doi: 10.3389/fmicb.2019.01014 (PMC6527751; doi:10.3389/fmicb.2019.01014)
Supplement: Supplementary file 5 [file Data_Sheet_1.docx]

**Supplementary Figure 1. Geographical location of Chile Bay:** The map shows the location of Greenwich Island, South Shetland Islands, on the West Antarctic Peninsula. The star indicates sampling site for surface seawater, sampled during February and March 2014.

**Supplementary Figure 2. Classification of the Pseudoalteromonas phage CBA:** Classification of the Query Phage in relative to other related Phages in Aclame (A classification of mobile genetic elements). Using the VIRFAM online platform, the predicted proteins from the genome of the novel Myovirus are compared with the Head-neck-tail module genes database. The Pp_CBA is clustered with the Type1 Myoviridae (Cluster 7).

**Supplementary Figure 3. Genomic structural comparison of the Pseudoalteromonas phage CBA and related virus:** Full genome alignment comparison with Mauve between the reported phage genome PpCBA, the Pseudoalteromonas phage H101 and representatives Felix O1 virus’s genomes are available in the databases. Corresponding aligning regions across genomes are grouped and colored by similarity.

**Supplementary Figure 4. Correlation between environmental parameters and the microbial community from Chile Bay:** Spearman correlation coefficients analyses, based on environmental factors (i.e.Temperature, Salinity, Nutrients) and the relative abundances and transcriptomic activity of the main phytoplankton, bacterioplankton and viral groups of the microbial community from L-C and H-C samples.

**Supplementary Table 1. Environmental parameters from Chile Bay**

**Supplementary Table 2. Total taxonomical classification and quality distribution of the metagenomic reads**

**Supplementary Table 3. Predicted proteins from novel Pseudoalteromonas phage CBA genome**

**Supplementary Table 4. Partial predicted proteins from novel Antarctic Phycodnavirus**
