# Supplementary figures and images for: Elucidating Viral Communities During a Phytoplankton Bloom on the West Antarctic Peninsula
Source: Front Microbiol. 2019 May 14;10:1014. doi: 10.3389/fmicb.2019.01014 (PMC6527751; doi:10.3389/fmicb.2019.01014)

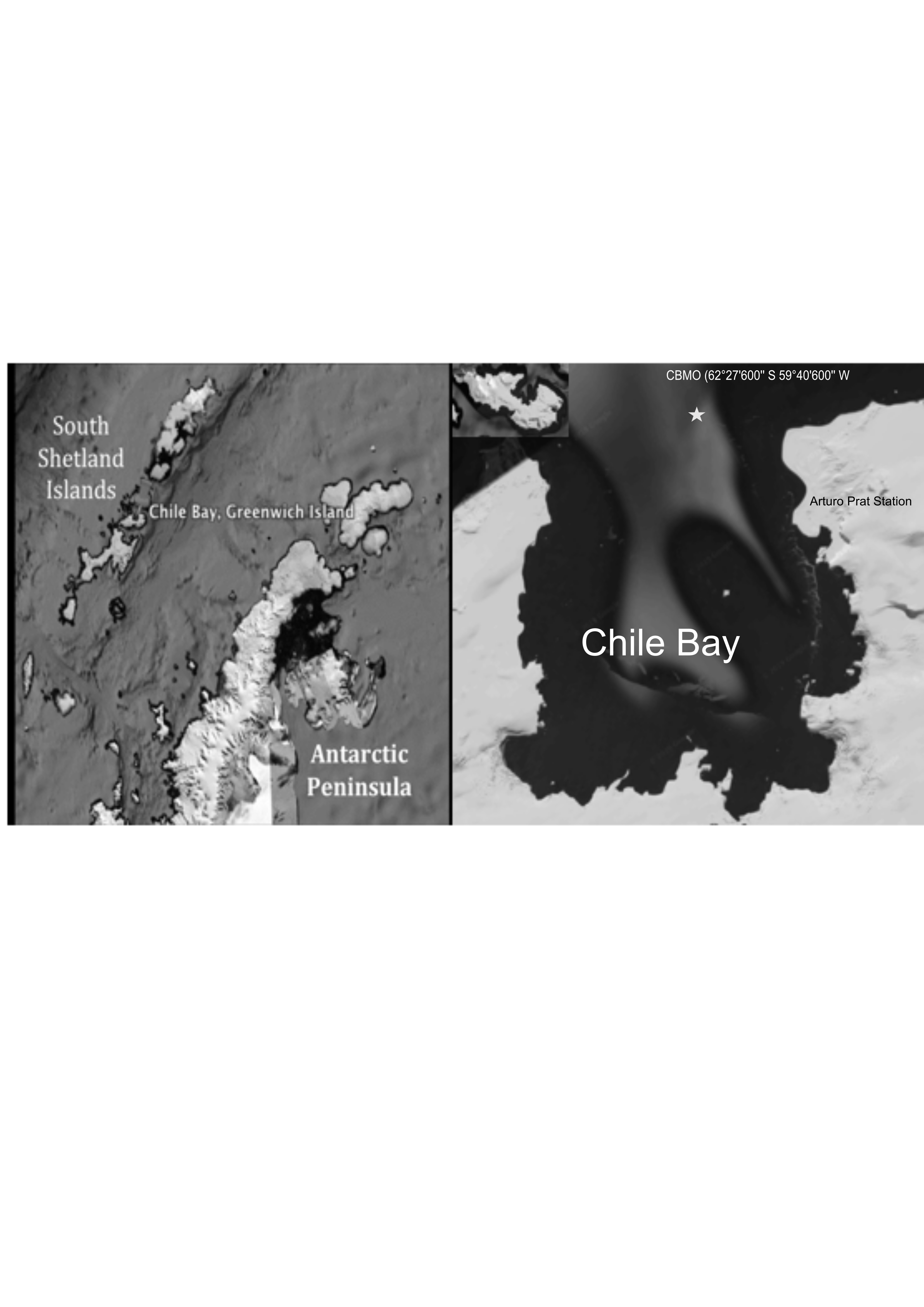

Supplement: Supplementary file 6 [file Image_1.TIFF]

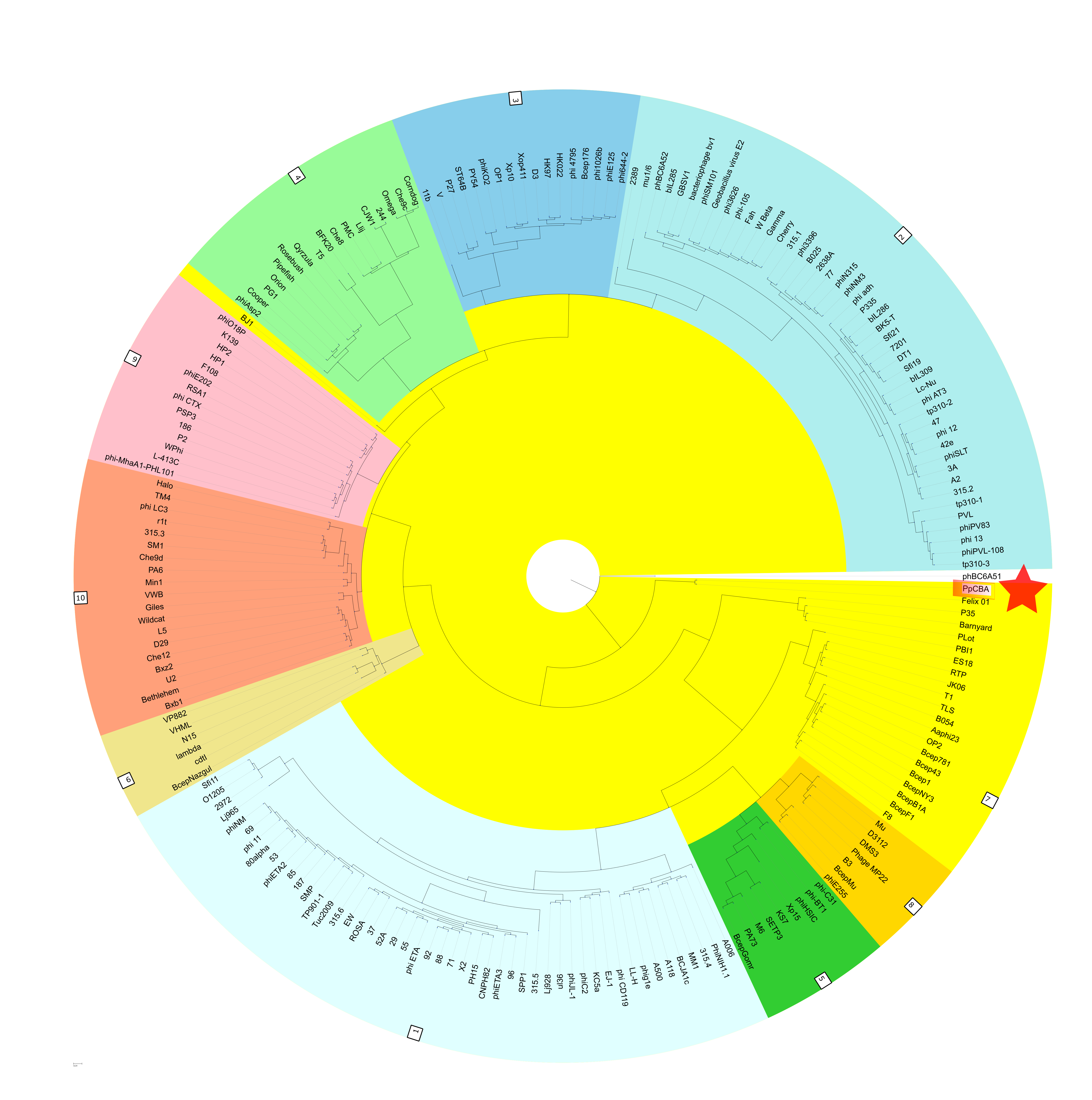

Supplement: Supplementary file 7 [file Image_2.TIFF]

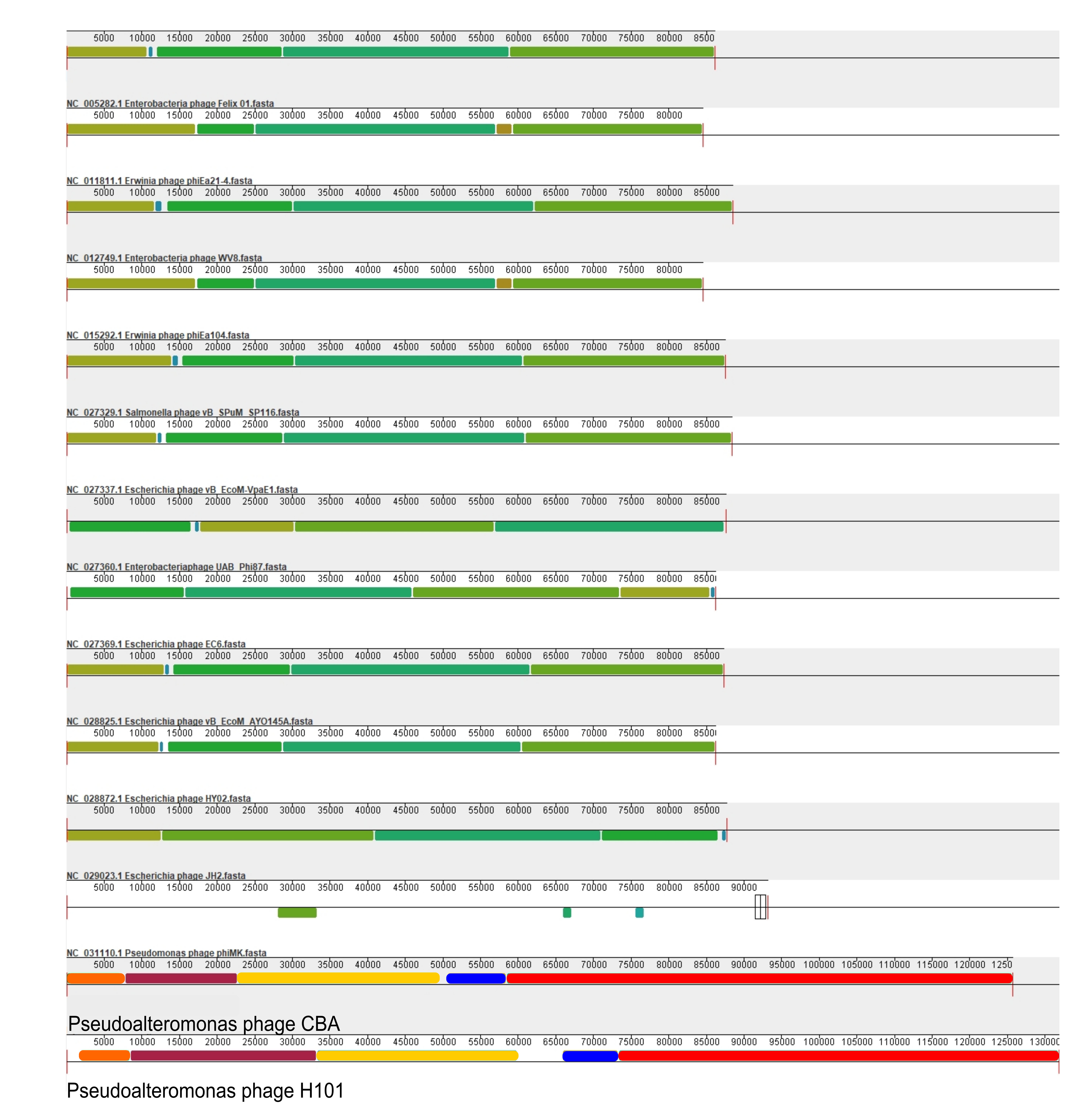

Supplement: Supplementary file 8 [file Image_3.TIFF]

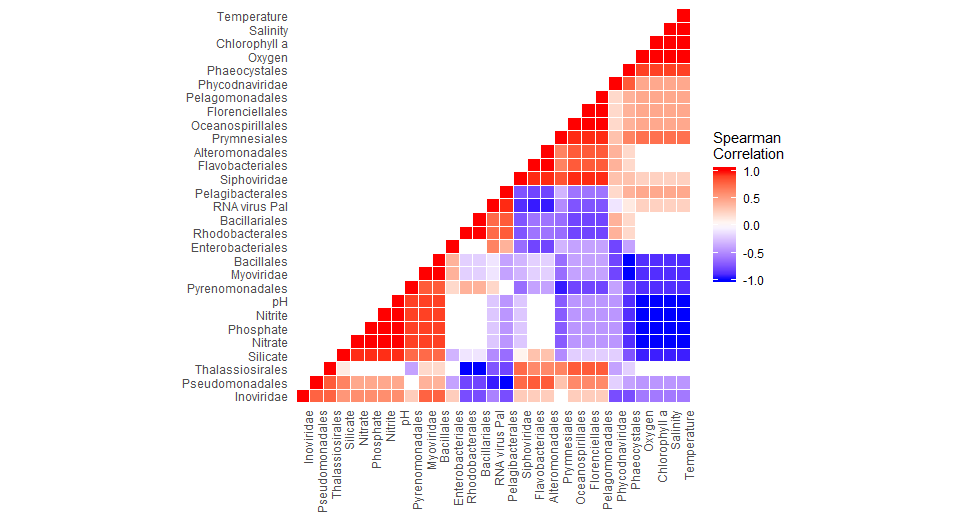

Supplement: Supplementary file 9 [file Image_4.TIFF]
